# The polyene antifungals, amphotericin B and nystatin, cause cell death in *Saccharomyces cerevisiae* by a distinct mechanism to amphibian-derived antimicrobial peptides

**DOI:** 10.1186/1476-0711-13-18

**Published:** 2014-05-12

**Authors:** George Serhan, Colin M Stack, Gabriel G Perrone, Charles Oliver Morton

**Affiliations:** 1 University of Western Sydney, School of Science and Health, Campbelltown Campus, Narellan Road, Campbelltown, NSW 2560, Australia

**Keywords:** Amphotericin B, Antifungal resistance, *YOL002C*, Antifungals

## Abstract

**Background:**

There is a pressing need to identify novel antifungal drug targets to aid in the therapy of life-threatening mycoses and overcome increasing drug resistance. Identifying specific mechanisms of action of membrane-interacting antimicrobial drugs on the model fungus *Saccharomyces cerevisiae* is one avenue towards addressing this issue. The *S. cerevisiae* deletion mutants *Δizh2*, *Δizh3*, *Δaif1* and *Δstm1* were demonstrated to be resistant to amphibian-derived antimicrobial peptides (AMPs). The purpose of this study was to examine whether AMPs and polyene antifungals have a similar mode of action; this was done by comparing the relative tolerance of the mutants listed above to both classes of antifungal.

**Findings:**

In support of previous findings on solid media it was shown that *Δizh2* and *Δizh3* mutants had increased resistance to both amphotericin B (1–2 μg ml^−1^) and nystatin (2.5 – 5 μg ml^−1^) in liquid culture, after acute exposure. However, *Δaif1* and *Δstm1* had wild-type levels of susceptibility to these polyenes. The generation of reactive oxygen species (ROS) after exposure to amphotericin B was also reduced in *Δizh2* and *Δizh3*. These data indicated that polyene antifungal and AMPs may act via distinct mechanisms of inducing cell death in *S. cerevisiae*.

**Conclusions:**

Further understanding of the mechanism(s) involved in causing cell death and the roles of *IZH2* and *IZH3* in drug susceptibility may help to inform improved drug design and treatment of fungal pathogens.

## Findings

The incidence of invasive fungal diseases is increasing, especially in those individuals undergoing immunosuppressive treatments or those with predisposing conditions [1]. The number of antifungal agents available is limited due to the biological overlap and conserved nature of cellular processes between fungi and mammalian cells. Antifungal drug resistance, particularly to azoles, is also increasing which has created a strong need for the identification of novel antifungal therapies and drug targets [2].

The major targets for antifungal drugs are the cell wall and plasma membrane. The major groups of antifungals in use today are the polyene (amphotericin B and nystatin) and azole (e.g. voriconazole) classes which target ergosterol in the fungal plasma membrane and ergosterol synthesis, respectively [3]. In fungi ergosterol serves an analogous function to cholesterol in mammalian cell membranes and forms the basis of the selective toxicity of many antifungals. Azoles target the proteins responsible for ergosterol biosynthesis whereas polyenes interact with ergosterol in the plasma membrane [4]. Resistance to azoles has been shown to result from nucleotide polymorphisms is the *cyp51a* gene whereas resistance to polyenes is less common due to it targeting an essential structural component of the membrane [5]. Polyenes primarily act by binding membrane ergosterol and forming pores in the plasma membrane which is similar to the proposed mode of action for antimicrobial peptides (AMPs). These are a group of peptides that form an important part of the innate immune system of animals and include the human defensins [6].

Detailed analysis of the activity of amphibian-derived AMPs indicated that they induced programmed cell death (PCD) in *Saccharomyces cerevisiae*[7] which was also observed during exposure of *Aspergillus fumigatus* to amphotericin B [8]. Formation of pores in membranes is now thought to be a secondary inhibitory mechanism for both of these classes of molecule with primary activity leading to intracellular oxidative damage and interaction with organelles [9]. The purpose of this study was to build on previous research that identified *S. cerevisiae* deletion mutants that were resistant to amphibian-derived AMPs and to examine if they are also resistant to amphotericin B. This may indicate similarities in the mode of action and induction of PCD between these antifungal agents.

The *S. cerevisiae* deletion mutants *Δizh2*, *Δizh3*, *Δstm1* and *Δaif1* were selected from the haploid yeast deletion mutant library (strain BY4742 from Thermo Scientific) based on the resistance of these mutants to amphibian-derived antimicrobial peptides [7]. Cultures of these strains and wild-type were grown in YPD broth (Sigma-Aldrich) at 30°C with shaking at 150 rpm overnight.

After overnight growth cultures were adjusted to an OD_600_ of 0.5 before treatment with 1–2 μg ml^−1^ amphotericin B (Sigma-Aldrich) or 2.5–5 μg ml^−1^ of nystatin (Sigma-Aldrich). These doses are greater than the minimum inhibitory concentrations of the drugs (0.1–2 μg ml^−1^ amphotericin B and 2.0 μg ml^−1^ nystatin) to test drug resistance. Drug-treated cultures were incubated for one, two and three hours after which serial dilutions of each culture were prepared and plated onto YPD agar; these were incubated at 30°C for two days. The plates were examined for growth and the numbers of colony forming units (CFU) were counted. Three independent experiments were conducted for each treatment.

Production of reactive oxygen species (ROS) was assessed to examine secondary effects of polyenes. Cultures were treated overnight with 0.5 or 1.0 μg ml^−1^ amphotericin B and ROS was detected by a previously described method using 2,7-dichlorofluorescein (Sigma Aldrich, St Louis, MO, USA) [7].

Colony forming units (CFU) data were analysed by repeated measures ANOVA comparing each deletion to the wild-type and Bonferroni’s multiple comparison to compare individual treatments. The ROS data was analysed by Mann–Whitney tests comparing treated to untreated cells for each strain.

### *Δizh2* and *Δizh3* exhibit increased resistance to polyene antifungals

**Figure 1:**
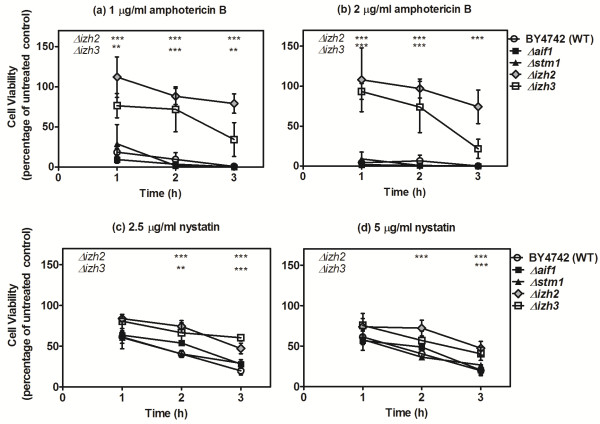
**The effects of exposure to polyene antifungals on the wild-type and selected deletion mutants of *Saccharomyces cerevisiae*.** The effect of drug was determined by expressing each treatment as a percentage of control growth to account for variations in starting cell numbers between treatments and experiments. **(a-b)***Saccharomyces cerevisiae* was exposed to two concentrations of amphotericin B; 1.0 μg ml^−1^ and 2.0 μg ml^−1^ for one, two and three hours. The number of viable cells reduced over time for each strain, however *Δizh2* and *Δizh3* were significantly more resistant than the wild-type and other strains tested at all doses. **(c-d)***S. cerevisiae* was exposed to two concentrations of nystatin; 2.5 μg ml^−1^ and 5.0 μg ml^−1^ for one, two and three hours. The number of viable cells reduced over time for each strain; however *Δizh2* and *Δizh3* were significantly more resistant than the other strains after two and three hour’s incubation. The data in a-d are means and standard deviations of three replicate experiments: these were analysed by repeated measures ANOVA and Bonferroni’s multiple comparison test; *p < 0.05, **p < 0.01, ***p < 0.001.

Incubation of wild-type *S. cerevisiae* cells with increasing doses of amphotericin B led to a reduction in viable cells from 80% viability in the wild type (BY4742) after 1 hour exposure at 1.0 μg ml^−1^ to 4% viability in the wild type after 1 hour exposure to 2.0 μg ml^−1^ of amphotericin B (Figure 1a and b). The absolute values equating to 100% cell viability were 1 × 10^5^ CFU ml^−1^ after 1 hour, 1.7 × 10^5^ CFU ml^−1^ after 2 hours and 3.7 × 10^5^ CFU ml^−1^ after 3 hours. The wild type, *Δaif1* and *Δstm1* mutants were highly sensitive to amphotericin B and nystatin with significant reductions in viability over time in a dose-dependent manner (Figure 1a-d). The *Δizh2* and *Δizh3* mutants showed increased resistance to exposure to amphotericin B, which was particularly pronounced at higher drug doses. The most pronounced differences in drug resistance were observed when these deletion mutants were exposed to amphotericin B.

**Figure 2:**
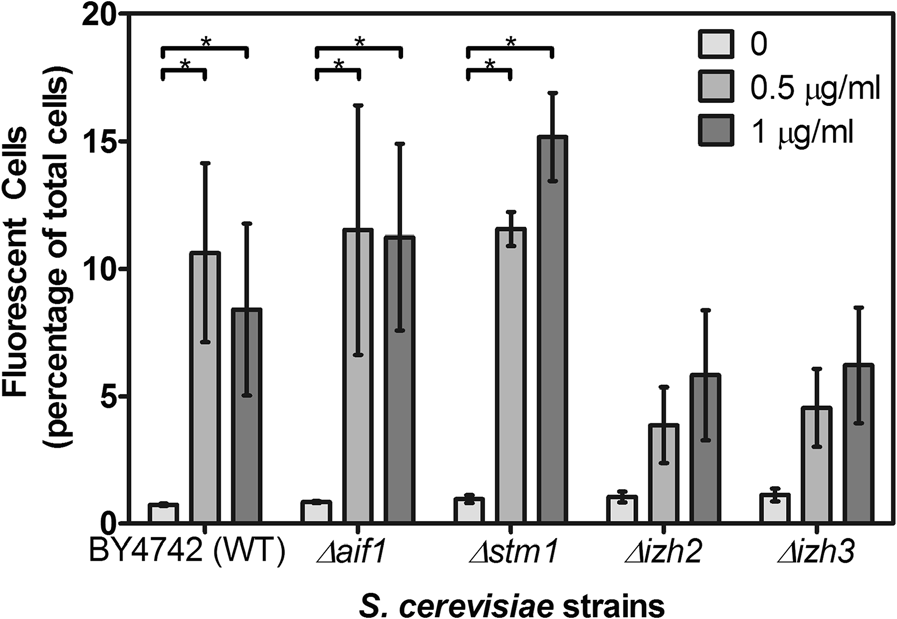
**The effect of prolonged (24 h) exposure to amphotericin B (0.5 μg ml**^**−1 **^**or 1.0 μg ml**^**−1**^**) on wild-type and selected deletion mutants of *S. cerevisiae *measured by the production of reactive oxygen species (ROS) within yeast cells.** Data presented are ratios of the number of fluorescent cells compared to the total population; at least 200 cells from ten fields of view were used to analyse each treatment. Incubation with amphotericin B induced significantly greater production of ROS in treated wild type BY4742, *Δaif1* and *Δstm1*. Amphotericin B had no significant effect on *Δizh2* and *Δizh3*. The data shown are means and standard deviations from three replicate experiments and were analysed by the Manny-Whitney test to compare treated samples with the untreated control; *p < 0.05, **p < 0.01, ***p < 0.001.

Prolonged exposure to amphotericin B resulted in formation of reaction oxygen species (ROS) in drug-treated *S. cerevisiae* cells, all strains (Figure 2). Amphotericin B treatment significantly increased the number of fluorescent cells induced in BY4742, *Δaif1* and *Δstm1*, whereas no significant increase was observed in *Δizh2* and *Δizh3*. ROS generation may indicate induction of PCD and correlates with the oxidative damage that has been linked to the activity of amphotericin B [10,11]. Similar data were obtained for the reaction of *S. cerevisiae* and mutants treated with 5.0 μg ml^−1^ nystatin (data not shown).

### Polyene antifungals appear to cause cell death in yeast by a distinct mechanism compared to AMPs

In this study we demonstrated that deletion of *IZH2* or *IZH3* conferred increased resistance to amphotericin B, which was previously reported for amphibian-derived AMPs [7]. AMPs [7] and polyenes (Figure 2) lead to intracellular accumulation of ROS that is associated with oxidative damage and PCD. However, susceptibility of *Δaif1* and *Δstm1* mutants to amphotericin B suggests that a different mechanism may be involved in polyene-mediated cell death. That both of these mechanisms are likely caspase-independent in *Aspergillus fumigatus*[8] but not in *Candida* Biofilms [12], which underlines the need to elucidate the variety of mechanisms leading to cell death in microbes in order to exploit these mechanisms as a means of controlling fungal infections.

The major similarity between the two classes of antifungal was the requirement for *IZH2* and *IZH3* for full susceptibility of *S. cerevisiae* to membrane-interacting antifungals. These genes are members of the progestin and adipoQ receptor (PAQR) family, which is a group of membrane embedded receptor molecules [13]. Previous studies had shown that deletion of *IZH2* and *IZH3* led to resistance to polyenes [14,15] and that the plant defence protein osmotin was shown to bind to Izh2p to induce PCD [16]. The effective drug doses in this study were greater than in the previous study which reported polyene resistance in *Δizh2* and *Δizh3*[15]. This may be attributed to different formulations of the drug and different culture conditions since other studies have tested drug effects using agar media. The proposed mechanism of action of osmotin, a homologue of adiponectin, is to bind specifically to the receptor domain of Izh2p which causes a signal cascade that triggers PCD [17]. It is possible that the polyenes nystatin and amphotericin B interact with izh2p or izh3p to induce PCD in *S. cerevisiae*; since the PAQR class of proteins is widely distributed in fungi this would provide a target for rational drug design.

The direct activation of a receptor by the selected polyenes may not be the mechanism of action since these antifungals are not structurally similar to osmotin which is a 24 kDa protein that is homologous to adiponectin [16]. The *IZH* family of genes has pleiotropic effects on gene expression and their cellular roles cannot yet be functionally assigned to a single biochemical pathway [18,19]. Along with being involved in zinc homeostasis this gene family is also associated with sterol metabolism [18,20]. Specifically, it is thought that their function in sterol metabolism may influence membrane permeability to alter ion homeostasis [19]. For example deletion of *IZH3* exhibits a positive genetic interaction with the genes *SCS7* (sphingolipid alpha-hydroxylase) [21,22] and *AUR1* (inositol phosphoceramide synthase) [23]. These are involved in the biosynthesis of sphingolipids which are essential components of the cell membrane. Deletion of *IZH3* may therefore alter membrane composition affecting the ability of polyenes to bind membrane ergosterol leading to enhanced resistance.

This study confirms the resistance of *Δizh3* and *Δizh2* to polyene antifungals and indicates that Izh3p may have functional similarities to Izh2p with regard to membrane ergosterol content. These data also suggest that polyenes and amphibian-derived antimicrobial peptides may induce PCD in *S. cerevisiae* by distinct mechanisms. A full understanding of mechanisms of cell death induced by these antifungal agents may yield an important advance in the development of new classes of antifungal drugs.

## Availability of supporting data

The data supporting the results of this study are included within this article.

## Abbreviations

YPD: Yeast extract peptone dextrose; IZH2/3: Implicated in zinc homeostasis; STM1: suppressor of ToM1; AIF1: Apoptosis inducing factor.

## Competing interests

The authors declare that they have no competing interests that could affect the integrity of this study.

## Authors’ contributions

GS performed the experimental work for the study. CS assisted in experimental design and preparation of the manuscript. GP assisted in experimental design, analysis and manuscript preparation. COM designed the study, assisted in data analysis and wrote the manuscript. All authors read and approved the final manuscript.
